# Intention to Use Mobile-Based Partograph and Its Predictors Among Obstetric Health Care Providers Working at Public Referral Hospitals in the Oromia Region of Ethiopia in 2022: Cross-Sectional Questionnaire Study

**DOI:** 10.2196/51601

**Published:** 2024-05-10

**Authors:** Kefyalew Naniye Tilahun, Jibril Bashir Adem, Wabi Temesgen Atinafu, Agmasie Damtew Walle, Nebyu Demeke Mengestie, Abraham Yeneneh Birhanu

**Affiliations:** 1 College of Medicine and Health Sciences Ambo University Ambo Ethiopia; 2 Department of Public Health Arsi University Asella Ethiopia; 3 Department of Health Informatics Mettu University Mettu Ethiopia; 4 Department of Health Informatics Institute of Public Health University of Gondar Gondar Ethiopia

**Keywords:** mobile-based partograph, mHealth, mobile health, cross-sectional, questionnaire, questionnaires, survey, surveys, modified TAM, technology acceptance model, intention to use, obstetric health care providers, Ethiopia, intent, intention, TAM, experience, experiences, attitude, attitudes, opinion, opinions, perception, perceptions, perspective, perspectives, acceptance, adoption, partograph, digital health, health technology, birth, women's health, obstetrics, obstetric, obstetric health care, labor monitoring

## Abstract

**Background:**

A partograph is a pictorial representation of the relationship between cervical dilatation and the time used to diagnose prolonged and obstructed labor. However, the utilization of paper-based partograph is low and it is prone to documentation errors, which can be avoided with the use of electronic partographs. There is only limited information on the proportion of intention to use mobile-based partographs and its predictors.

**Objective:**

The objective of this study was to determine the proportion of obstetric health care providers at public referral hospitals in Oromia, Ethiopia, in 2022 who had the intention to use mobile-based partographs and to determine the predictors of their intention to use mobile-based partographs.

**Methods:**

We performed an institution-based cross-sectional study from June 1 to July 1, 2022. Census was conducted on 649 participants. A self-administered structured English questionnaire was used, and a 5% pretest was performed. Data were entered into EpiData version 4.6 and exported to SPSS version 25 for descriptive analysis and AMOS (analysis of moment structure; version 23) for structural and measurement model assessment. Descriptive and structural equation modeling analyses were performed. The hypotheses developed based on a modified Technology Acceptance Model were tested using path coefficients and *P* values <.05.

**Results:**

About 65.7% (414/630; 95% CI 61.9%-69.4%) of the participants intended to use mobile-based electronic partographs, with a 97% (630/649) response rate. Perceived usefulness had a positive influence on intention to use (β=.184; *P*=.02) and attitude (β=.521; *P*=.002). Perceived ease of use had a positive influence on attitude (β=.382; *P*=.003), perceived usefulness (β=.503; *P*=.002), and intention to use (β=.369; *P*=.001). Job relevance had a positive influence on perceived usefulness (β=.408; *P*=.001) and intention to use (β=.185; *P*=.008). Attitude positively influenced intention to use (β=.309; *P*=.002). Subjective norms did not have a significant influence on perceived usefulness (β=.020; *P*=.61) and intention to use (β=–.066; *P*=.07).

**Conclusions:**

Two-thirds of the obstetric health care providers in our study intended to use mobile-based partographs. Perceived usefulness, perceived ease of use, job relevance, and attitude positively and significantly influenced their intention to use mobile-based electronic partographs. The development of a user-friendly mobile-based partograph that meets job and user expectations can enhance the intention to use.

## Introduction

### Background

Globally, maternal mortality remains a persistent and potentially preventable issue of great concern. In 2020, every 2 minutes, a woman died due to pregnancy-related preventable causes, indicating that about 800 women died every day, resulting in a maternal mortality ratio of 223 deaths per 100,000 live births [1]. By lessening maternal mortality to roughly 70 per 100,000 live births between 2016 and 2030, the Sustainable Development Goal initiatives hope to avert the maternal mortality rates [2]. Contrary to goals set according to the Sustainable Development Goals 3.1, the global maternal mortality rate increased from 151 in 2019 to 152 per 100,000 live births in 2020 [3]. In Ethiopia, maternal death rate was 412 per 100,000 live births in 2016 [4]. It stayed high, accounting for 412 per 100,000 live births in 2019 [5]. In Ethiopia, prolonged and obstructed labor account for 22% of all maternal deaths [6]. Although prolonged and obstructed labors are among the leading causes of death in resource-poor settings, they can be diagnosed and averted with correct partograph use [7,8]. A partograph is a graphic representation of the labor’s progress that includes pertinent information about the mother and the fetus [9]. In this regard, one of the most important ways in assuring high-quality care for both the mother and the newborn during labor is to use the partograph [10].

Despite its significance, partograph use by obstetric health care providers is still low in Ethiopia [6,7,11-15]. In addition to this, the paper-based approach is prone to recording errors due to health care providers’ overburdened and retrospective data entry [16]. Paper-based partographs are also exposed to incomplete reporting of parameters. In Uganda, only 24.6% of the partograph parameters demonstrated complete details [17]. In a study in Jigjiga and Degehabur, about 64% of the partograph characteristics were only partially recorded [11]. A study in the West Shoa zone revealed that only 3% of the partographs examined was recorded according to the standard [7]. The rapid progress of technology is one of the many drivers now impacting health care systems [18], and offering health care services via mobile devices is now seen as a promising technological advancement [19]. The widespread availability of smartphones and tablets provides an opportunity for the use of a well-designed electronic partograph [16]. Digitizing the partograph improves adherence and overcomes the limitation of paper-based partographs [10,16,20-22]. The electronic partograph is a contemporary instrument for capturing labor data in real time, which can improve mother and infant outcomes [23]. Electronic partographs make it possible to improve the labor management system’s record of labor progress statistics and care given to mother and fetus, especially in low-income nations [10,20,24,25]. Electronic partographs also result in a significant reduction in the rate of prolonged labor from 42% to 29% and have a far greater usage rate than paper-based partographs [21]. In addition, electronic partographs are preferred over paper-based partographs by clinicians due to their ease of use and less time, improved performance, decreased referral rates, assured prompt referral when necessary, facilitation of reporting obligations, and enhancement of service quality [26,27].

Studies show that using mobile-based health services in the health sector have the potential to increment health service access, quality, adherence, and efficiency [28-35]. However, the technological benefit obtained depends on the rate of use and adherence of users. Human activities mainly depend on their behavioral intention, and the intention to use digital tools is a determinant factor of actual user behavior. Therefore, determining the behavioral intention to use and its predictors before the adoption of technology is important and prevents implementation failure [36]. Behavioral intention is the degree to which a person has made intentional plans to engage or abstain from engaging in a specific future conduct [37]. To facilitate future implementation, it is vital to ascertain the degree of intention to employ any digital tools in the health sector [36]. A variety of Technology Acceptance Models (TAMs) have been applied to identify and predict end user behavioral intention to utilize technology. Among these, Davis’s TAM is significant and effective at predicting users’ intent to use [38].

To increase understanding of the factors affecting behavioral intention, different scholars have modified the original TAM [39-45]. Therefore, we modified the TAM to increase the understanding of the predictors that influence the behavioral intention of obstetric health care providers to use mobile-based partographs because information on the proportion of intention to use mobile-based partographs and its predictors is limited. This study was intended to fill this gap.

### Theoretical Model and Hypothesis Development

TAM is the most well-known methodology for establishing and evaluating each person’s intent to embrace new technology [46]. It is a commonly used model that is used to anticipate possible users’ behavioral intentions to use a technological innovation [47]. TAM had been altered, nevertheless, to boost its capacity for predicting variations in usage intention. Studies on doctors’ approval of digital personal aids in Turkey [48], smart health care services among medical practitioners in China [49], health information system acceptance by hospital personnel in Greece [50], intention to use technology to attend to clients by health professionals in Ghana [51], and sustainable adoption of eHealth systems by health care professionals in Ethiopia [39] by using modified TAM report about 71%,71.5%, 87%, 97%, and 56% of variance in intention to use, respectively.

TAM was initially established with 2 key dimensions termed as perceived utility and perceived ease of use to identify the potential drivers of intention. The extent to which a person thinks using a given system would improve his or her performance at work is known as perceived usefulness [38]. According to studies [18,38,39,48,52] in health care settings, perceived usefulness had a significant and positive influence on intention to use. Perceived ease of use is another factor that establishes the end users’ behavioral intentions. The degree to which someone perceives a system to be simple to use is known as perceived ease of use [38]. The greater the user’s propensity to use something, the friendlier is the user experience [53]. Perceived ease of use significantly and positively influences intention to use [39,48,50,54] and end users’ attitudes toward using a particular approach or technology [55]. The attitude is described as an individual’s impression of the positive or negative implications of embracing technology [56]. Attitude toward using positively and significantly influences intention to use [52,57,58] and is influenced by perceived usefulness [58,59] and ease of use [39].

**Figure 1:**
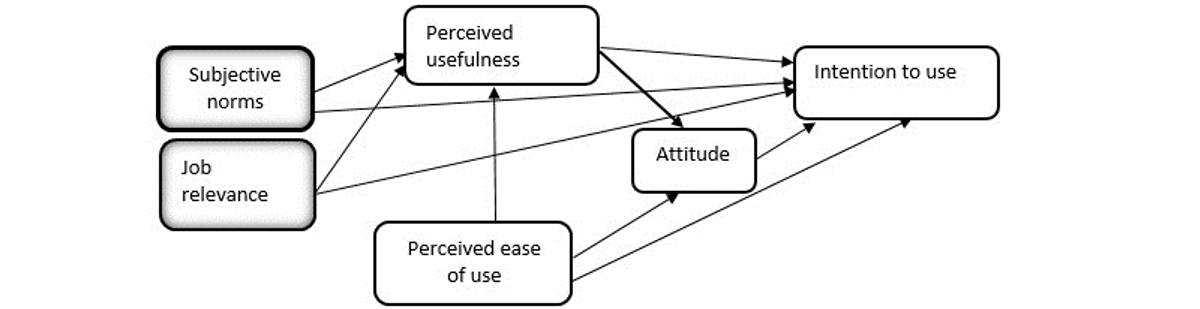
Our proposed modified Technology Acceptance Model based on the original Technology Acceptance Model of Davis et al [69].

According to the core construct of TAM, it is possible to add antecedents to improve the predictive power and understand the potential factors influencing behavioral intention. To this end, subjective norms and job relevance are important predictors added to the original TAM. Subjective norms refer to “a person’s view that the majority of influential individuals in his life believe he should or should not engage in the behavior in question” [60]. Studies have shown that subjective norms influence perceived usefulness [61] and intention to use [49,62]. The perception of whether a technology is suitable for their jobs can also have an impact on whether they intend to use it. Job relevance examines how users view using the system for work and is found to influence perceived usefulness and intention to use [44,45,63]. Whether the specified concept will mediate the link between constructs is another key factor to consider. The mediators act as a channel for latent concept effects to reach the dependent variables [64]. Perceived usefulness [65,66] and attitude [53,67,68] have been reported to act as mediators in TAM studies. The following hypotheses are formed in light of the information presented above (Figure 1).

Hypothesis 1: Perceived usefulness will have a positive effect on the intention to use mobile-based partographs.

Hypothesis 2: Perceived usefulness will have a positive effect on obstetric health care providers’ attitudes toward mobile-based partographs.

Hypothesis 3: Perceived ease of use will have a positive effect on obstetric health care providers’ attitudes toward mobile-based partographs.

Hypothesis 4: Perceived ease of use will have a positive effect on the perceived usefulness of mobile-based partographs.

Hypothesis 5: Perceived ease of use will have a positive influence on the intention to use mobile-based partographs.

Hypothesis 6: Obstetric health care providers’ attitudes toward using mobile-based partographs will positively influence intention to use.

Hypothesis 7: Job relevance will have a positive effect on the intention to use mobile-based partographs.

Hypothesis 8: Job relevance will have a positive effect on the perceived usefulness of mobile-based electronic partographs.

Hypothesis 9: Subjective norms will have a positive effect on the perceived usefulness of mobile-based partographs.

Hypothesis 10: Subjective norms will have a positive effect on the intention to use mobile-based partographs.

Hypothesis 11: Perceived usefulness mediates the relationship between job relevance and intention to use.

Hypothesis 12: Perceived usefulness mediates the relationship between subjective norms and intention to use.

Hypothesis 13: Perceived usefulness mediates the relationship between perceived ease of use and intention to use.

Hypothesis 14: Attitude mediates the relationship between perceived usefulness and intention to use.

Hypothesis 15: Attitude mediates the relationship between perceived ease of use and intention to use.

## Methods

### Study Design and Setting

An institution-based cross-sectional study was conducted from June 1 to July 1, 2022, at public referral hospitals in the Oromia region of Ethiopia. There are 30 municipal administrations and 23 zones in the Oromia region. Thirteen public referral hospitals, 33 general hospitals, 67 primary hospitals, 1383 health centers, and 6797 health posts are located in the Oromia region. In the 13 public referral hospitals, there were about 649 obstetric health care professionals employed. The midwives, nurses, integrated emergency obstetrics and surgery professionals, general practitioners, obstetricians, and gynecologists are trained obstetric health care professionals.

### Study Participants and Sample Size Determination

All obstetric health care providers who were working at Oromia region public referral hospitals and were available at the time of data collection were the source and study population of this study. The sample size for this study was calculated based on the rule of thumb of structural equation modeling. The most widely used rule of thumb is that for 1 free parameter, 10 observations are required [70,71]. The proposed model of this study had 55 free parameters. Therefore, n (number of free parameters × 10; 55 × 10) = 550, where n represents the sample size. Considering a 10% nonresponse rate, the final minimum sample size was 605.

### Sampling Procedure

In the 13 public referral hospitals of the Oromia region, there were 649 obstetric health care providers. From the onset, this study required a large sample size to test the developed hypothesis using the maximum likelihood estimator of the structural equation model. However, the number of study participants involved in this study was not adequate for sampling. For this reason, a census was conducted.

### Study Variables

The outcome variable of this study was the intention to use mobile-based partograph, whereas the mediator variables were attitude toward using and perceived usefulness. The independent variables of this study included technology acceptance–related exogenous latent variables (perceived ease of use, subjective norm, and job relevance), sociodemographic and other related factors of obstetric health care providers (age, sex, marital status, religion, profession, qualification, and years of experience), access to mobile devices, partograph learning, in-service training, and computer courses.

### Operational Definition

Behavioral intention is the extent to which an individual has made intentional plans to engage in or refrain from engaging in a specific future conduct [37]. The intention to use, in this case, refers to the likelihood of obstetric health care providers whether they intended to use mobile-based partograph if they will be offered. The construct had 4 items, and each was measured with a 5-point Likert scale response. The median score was used as a cutoff point. The obstetric health care provider who scored median and above on intention to use construct was considered as intended to use a mobile-based partograph otherwise unintended.

### Data Collection and Procedure

A self-administered structured English questionnaire was used to collect data. Regarding the latent variable, the questionnaire was adapted from different literatures [44,53,63,72-77]. The adapted questionnaire was modified to fit the context of this study. The structured questionnaire had 4 parts: the first part included the sociodemographic characteristics of the obstetric health care providers, the second part related to access to mobile devices, the third part related to partograph and computer courses, and the fourth part included technology acceptance–related parameters (perceived usefulness, perceived ease of use, intention to use, attitude, job relevance, and subjective norm). A total of 21 items were used in this study to test the proposed hypothesis. A Likert scale ranging from strongly disagree (1) to strongly agree (5) was used to rate the level of participant agreement toward the prepared close-ended questions.

### Data Quality Assurance

A pretest was done on 5% of the sample size among obstetric health care providers who were working out of the study area. One day of training was given to 4 data collectors and 4 supervisors on the objective of the study, data collection procedures, data confidentiality, and respondents’ rights. The data collectors were BSc graduates: 3 midwives, 2 health officers, and 3 nurses. Supervision was continuous and made by the supervisors and principal investigator throughout the data collection. After data collection, completeness was checked.

### Data Processing and Analysis

Before data analysis, the coded data were entered into EpiData (version 4.6) and finally exported into SPSS (version 25; IBM Corp) for descriptive analysis and AMOS (analysis of moment structure; version 23) for structural and measurement model assessment. Structural equation modeling is a multivariate statistical analysis technique that is used to analyze structural relationships. The data set was checked for missing values, and there were no missing data. The sociodemographic data were analyzed descriptively using SPSS, and the results were presented using a frequency table. Descriptive statistics was used to compute the proportion of intention to use mobile-based partographs, and the result was presented using the bar graph. The maximum likelihood estimation method was considered, and the assumption was checked. One assumption is the presence of multiple measurements for a construct. Perceived ease of use, perceived usefulness, and intention to use each have 4 items. However, attitude, subjective norms, and job relevance each have 3 items. Multicollinearity among the independent variables was assessed using the variance inflation factor. The result obtained (variance inflation factor ranged from 1.6 to 2.027) proved that there was no multicollinearity among independent variables.

Another assumption was univariate normality, which was assessed using kurtosis and skewness values, and the result shows there was univariate normality. The kurtosis value of less than 5, a critical ratio between –1.96 and +1.96, was used to declare the presence of multivariate normality. Unfortunately, the assumption of multivariate normality was not fulfilled. Therefore, bootstrapping technique was used to manage multivariate nonnormality.

Confirmatory factor analysis was used to perform measurement model assessment. Construct reliability was tested using Cronbach α and composite reliability. A cutoff point greater than .7 was used to declare the presence of internal consistency of the item that measured construct [78]. The recommended Cronbach α value should be .7 and above [79,80]. Furthermore, the composite reliability should be greater than .7 [36]. The average variance extracted and factor loading was used to measure convergent validity. In the measurement model assessment, the average variance extracted value greater than 0.50 [45,64] and factor loading of at least 0.6 [75] should be used to establish convergent validity. Discriminant validity assesses the distinctness of construct when measured by their respective items. The discriminant validity was determined using the square root of average variance extracted, and the value should be greater than the interconstruct correlations to declare whether the discriminant validity of the construct was achieved [64]. The degree to which one construct differs from every other construct in the instrument is indicated by its discriminant validity [81].

Model goodness of fit was checked both for measurement and structural model assessment. A model fit index of the ratio of chi-square to degrees of freedom ≤3 [36], comparative fit index >0.90, goodness-of-fit index >0.90, adjusted goodness-of-fit index >0.85, normalized fit index >0.90, standardized root mean square residual <0.08, and root means square error of approximation <0.008 index value were used to measure and declare the model’s goodness of fit [39,50]. We planned to perform model modification to improve the model fitness if the initial model did not fit by deleting the factor loading value with<0.5 covariate error terms [82]. In this regard, even if the measurement model fitness was achieved initially, the model modification was performed since the chi-square to the degree of freedom for the structural model was 3.128. Therefore, to increase the model fitness, we covariate the error term 15 and 16 on the intention to use the latent variable. Finally, the overall model fitted the data well.

After measurement model assessment, structural model fitness was checked and the model fit the data well. Then, the structural model assessment was performed. Based on AMOS output, the standardized path coefficient and the level of significance were used to test the developed hypothesis and determine the association between the latent variables of the study. The standardized regression weights showed the strength of association between latent variables [83], and *P* values less than .05 showed the level of significance considered. The square multiple correlations were used to report the proportion of variances in endogenous latent variables explained by exogenous variables. The bootstrap method was used to test the mediation effect.

### Ethics Approval

Ethical clearance was obtained from the University of Gondar, and this study was approved by its ethics review board (Ref/IPH/2129/2014). A letter of support was obtained from the Department of Health Informatics, and written consent was taken from each study participant.

## Results

### Sociodemographic Characteristics of the Participants

A total of 649 participants were planned to be included in this study from all public referral hospitals in the Oromia region for the assessment of their intention to use mobile-based partographs and the predictors for their intention to use. Among them, 97% (630/649) gave their consent and completed the questionnaire. The results of this study show that almost more than half (344/630, 54.6%) of the study participants were males, while 45.4% (286/630) of the participants were females. The median age of the study participants was 32 (IQR 9) years. The majority of the participants were in the age group of 30-39 years. About 34.4% (217/630) of the respondents were Orthodox Christians in religion. Among the study participants, 371 (58.9%) respondents were married and 218 (36.4%) were single. Regarding their profession, more than half (351/630, 55.7%) were midwives, 96 (15.2%) were nurses, and 95 (15.1%) were general practitioners. Of the total study participants, 497 (78.9%) were bachelor’s degree holders and 78 (12.5%) were a specialist in their qualifications. Almost half (328/630, 52.1%) of the study participants had ≤4 years of working experience with the qualification they had. All the sociodemographic characteristics of the study participants are shown in Table 1.

**Table 1:** Sociodemographic characteristics of the obstetric health care providers who were working at public referral hospitals in the Oromia region of Ethiopia in 2022 (N=630).

Demographic characteristics			Values, n (%)
**Sex**			
	Male	344 (54.6)	
	Female	286 (45.4)	
**Age (years)**			
	20-29	232 (36.8)	
	30-39	299 (47.5)	
	>40	99 (15.7)	
**Religion**			
	Orthodox Christian	217 (34.4)	
	Muslim	167 (26.5)	
	Protestant	208 (33)	
	Others^a^	38 (6)	
**Marital status**			
	Married	371 (58.9)	
	Single	218 (34.6)	
	Divorced	25 (4)	
	Separated	10 (1.6)	
	Widowed	2 (0.3)	
	Others^b^	4 (0.6)	
**Profession**			
	Midwives	351 (55.7)	
	Nurses	96 (15.2)	
	General Practitioner	95 (15.1)	
	Obstetrician and Gynecologist	78 (12.4)	
	Others^c^	10 (1.6)	
**Level of qualification**			
	Bachelors	497 (78.9)	
	Masters	48 (7.6)	
	Specialists	79 (12.5)	
	Others^d^	6 (1)	
**Years of working experience**			
	≤4	328 (52)	
	5-9	195 (31)	
	≥10	107 (17)	

^a^Waaqeffanna, Adventist, and Catholic.

^b^In a relationship.

^c^Integrated Emergency Obstetric Surgery professionals.

^d^Diploma.

### Access to Mobile Devices and Partographs

In this study, of the 630 participants, 625 (99.2%) had access to mobile devices (Table 2). Approximately 93.1% (582/630) of the obstetric health care providers had access to a smartphone. About 77.8% (490/630) of the study participants had a partograph. However, only 326 (51.7%) study participants had taken in-service training for using paper-based partographs. Regarding the computer course, 310 (80.7%) took basic computer training. Table 2 shows the frequency of access to mobile devices, partograph learning, partograph in-service training, and computer courses.

**Table 2:** Access to mobile devices, partograph training, and computer courses by obstetric health care providers who were working at the public referral hospitals in the Oromia region of Ethiopia in 2022 (N=630).

Variables			Values, n (%)
**Access to mobile devices**			
	Yes	625 (99.2)	
	No	5 (0.8)	
**Mobile device type**			
	Smartphone	582 (93.1)	
	Tablet	33 (5.3)	
	Others^a^	10 (1.6)	
**Study partograph**			
	Yes	490 (77.8)	
	No	140 (22.2)	
**In-service training on paper-based partograph**			
	Yes	326 (51.7)	
	No	304 (48.3)	
**Computer courses**			
	Yes	384 (61)	
	No	246 (39)	
**Computer course level**			
	Basic course	310 (80.7)	
	Advanced training	74 (19.3)	

^a^Basic phones or feature phone.

### Intention to Use Mobile-Based Partographs

Among the 630 obstetric health care providers, 414 had intention to use mobile-based partographs. Thus, about 65.7% (414/630; 95% CI 61.9%-69.4%) or two-thirds of the study participants scored median and above of intention to use. The median score of intention to use mobile-based partograph was 16 (IQR 1.5). The minimum and maximum scores for intention to use were 4 and 20, respectively. Figure S1 of Multimedia Appendix 1 shows the proportion of intended and unintended use of mobile-based electronic partographs among obstetric health care providers who were working at public referral hospitals in the Oromia region in 2022. In this study, Cronbach α and composite reliability values were greater than .9. All the factor loading values were found in the range of 0.843 to 0.946, and the average variance extracted value was found in the range of 0.761 to 0.840. Hence, construct reliability and convergent validity of the measurement model were achieved.

### Discriminant Validity

The finding of this study indicates that the square root of the average variance extracted value was greater than the value of the interconstruct correlations. Therefore, the discriminant validity of the measurement model was achieved. Table S2 of Multimedia Appendix 1 demonstrates the discriminant validity of the model. The bold values in the table represent the square root of the average variance extracted.

### Measurement Indices of the Goodness of Fit of Model

In this study, all the obtained values of the measurement model fit indices were in an acceptable range. Hence, the result of this study indicated that the modified proposed model fitted the data well. Table 3 shows the result of model fit indices [36,39,50].

**Table 3:** Results of the indices of goodness of fit of the measurement model assessment.

Model fit indices, citation	Cutoff point	Result obtained^a^	Fit decision
Chi-square to degree of freedom [36]	≤3	2.245	Accepted
Goodness-of-fit index [50]	>0.9	0.945	Accepted
Adjusted goodness-of-fit index [50]	>0.85	0.927	Accepted
Normed fit index [50]	>0.9	0.974	Accepted
Comparative fit index [39,50]	>0.9	0.985	Accepted
Root mean square residuals [39,50]	<0.08	0.022	Accepted
Root mean square error of approximation [50]	<0.08	0.044	Accepted

^a^The result is obtained from the measurement model assessment using AMOS (analysis of moment structure; version 23) software to check model fitness.

### Structural Model Assessment

Among the 10 proposed hypotheses of the direct relationship, 8 were supported by the collected data. However, hypothesis 9 and hypothesis 10 failed to support the proposed hypothesis. Our study shows that perceived usefulness positively influences intention to use (β=.184; *P*=.02). In addition, perceived usefulness had a positive influence on attitude (β=.512; *P*=.002). According to our finding, perceived ease of use positively influenced attitude (β=.382; *P*=.003), perceived usefulness (β=.503; *P*=.002), and intention to use (β=.369; *P*=.001). Perceived ease of use had the strongest path coefficient in influencing intention to use mobile-based partographs.

Job relevance in the structural model had a positive and significant influence (β=.408; *P*=.002) on perceived usefulness. In addition, job relevance (β=.185; *P*=.008) positively and significantly influenced the intention to use. The results of this study also indicated that attitude (β=.309; *P*=.002) positively and significantly influenced intention to use.

**Figure 2:**
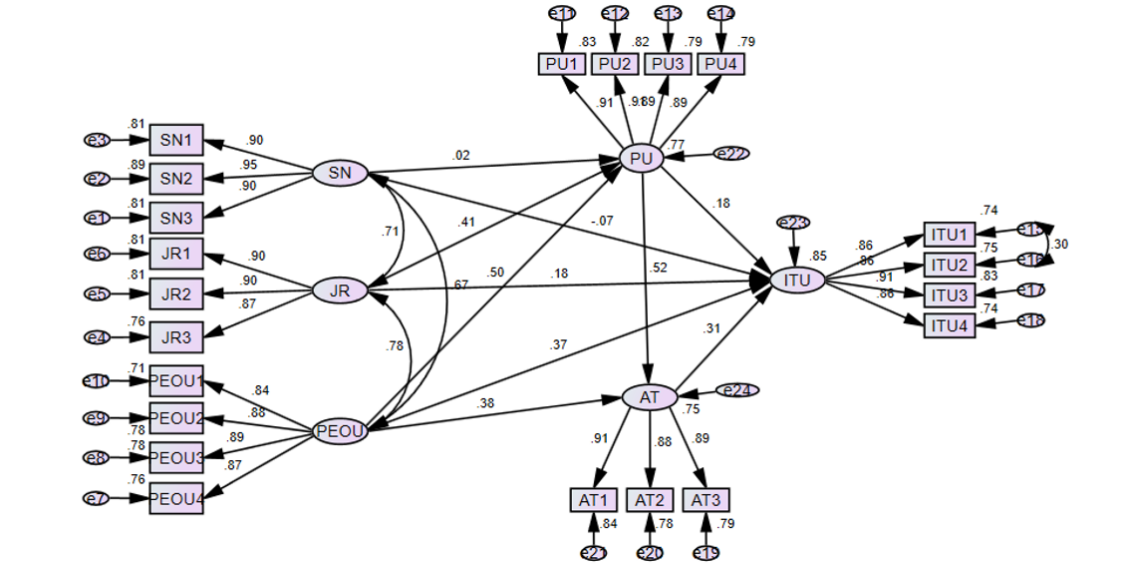
AMOS (analysis of moment structure; version 23) output of the standardized estimate. AT: attitude; ITU: intention to use; JR: job relevance; PEOU: perceived ease of use; PU: perceived usefulness; SN: subjective norms.

Subjective norms had insignificant influences on perceived usefulness (β=.02; *P*=.61) and intention to use (β=–.066; *P*=.07). Therefore, hypotheses 9 and 10 failed to support the developed hypothesis. Perceived ease of use, attitude, and job relevance had a 0.369, 0.309, and 0.185 path coefficient in association with intention to use, respectively. Results from the AMOS output of the proposed model showed that perceived usefulness was influenced by both job relevance and perceived ease of use (Figure 2). However, perceived ease of use (β=.503) had higher path coefficient than job relevance (β=.408) in influencing perceived usefulness. Perceived usefulness and perceived ease of use influenced the attitude toward using electronic partographs. Perceived usefulness (β=.521) had a higher path coefficient than perceived ease of use (β=.382) in influencing attitude toward using electronic partographs. Table 4 shows the results of the hypothesis testing of the direct path analysis of the proposed model.

**Table 4:** The results of the hypothesis testing of the proposed model.

Hypothesis	Causal path	β (path coefficient)	Critical ratio	*P* value	Decision
1	ITU^a^←PU^b^	.184	3.294	.02	Supported
2	AT^c^←PU	.521	9.918	.002	Supported
3	AT←PEOU^d^	.382	7.279	.003	Supported
4	PU←PEOU	.503	11.097	.002	Supported
5	ITU←PEOU	.369	7.160	.001	Supported
6	ITU←AT	.309	6.351	.002	Supported
7	ITU←JR^e^	.185	3.969	.008	Supported
8	PU←JR	.408	8.476	.002	Supported
9	PU←SN^f^	.020	0.540	.61	Not supported
10	ITU←SN	–.066	–2.060	.07	Not supported

^a^ITU: intention to use.

^b^PU: perceived usefulness.

^c^AT: attitude.

^d^PEOU: perceived ease of use.

^e^JR: job relevance.

^f^SN: subjective norms.

Among the 15 developed hypotheses, 5 of them deal with the mediation effect of perceived usefulness and attitude between the exogenous latent variables and outcome variable in this study. Table S3 of Multimedia Appendix 1 shows the result of the mediation analysis using the bootstrapping method. The 95% biased confidence interval and *P* value level were used to test the presence of a mediation. Our results showed that perceived usefulness partially mediates between job relevance and intention to use. Hence, hypothesis 11 was supported. However, perceived usefulness did not mediate the relationship between subjective norms and intention to use. Therefore, hypothesis 12 was not supported. However, perceived usefulness partially mediated perceived ease of use and intention to use. Therefore, hypothesis 13 was supported. Furthermore, attitude partially mediated the relationship between perceived usefulness and intention to use as well as perceived ease of use and intention to use. Consequently, hypothesis 14 and hypothesis 15 were supported. Table S3 of Multimedia Appendix 1 illustrates the result of the mediation analysis.

The squared multiple correlations indicate the predictive power of the model. In this study, the proposed model explained 85% of the variance in intention to use a mobile-based partograph. Perceived ease of use and job relevance aggregately explained 77% of the variance in perceived usefulness. However, perceived ease of use and perceived usefulness explained about 75% of the variance in attitude toward use. Table S4 of Multimedia Appendix 1 shows the result of the predictive power of the proposed model.

## Discussion

### Principal Findings

This study examines the proportion of obstetric health care providers in the Oromia region of Ethiopia who had the intention to use mobile-based partographs and the predictors for their intention to use. The result of our study revealed that about two-thirds (414/630, 65.7%) of the obstetric health care providers had the intention to use mobile-based partographs. Even if no similar study was conducted on the intention to use a mobile-based partograph, ascertaining the end user’s level of acceptance to use technology before execution serves as a prerequisite to judging the accomplishment of the execution [39]. In this regard, a study in north Gondar reported that 44% of the obstetric health care providers were willing to use mobile-based partographs [84]. The findings of [84] assist our study in promoting the execution of mobile-based partographs in clinical settings for enhancing the care given during the management of labor.

The results of our study regarding TAM were in line with those of the original TAM [69]. In this study, perceived ease of use (β=.503; *P*=.002) significantly and positively influenced the perceived usefulness of mobile-based partographs by obstetric health care providers. This study’s finding is in line with a study in Omaha on telemedicine acceptance (β=.56) [18] and that in Ethiopia on eHealth acceptance (β=.385) [39]. Further, this study finding is supported by a study conducted in Tanzania in which skilled birth attendants found that electronic partograph was useful, easy to use, and improved the quality of care [24]. This indicates that obstetric health care providers who perceive that mobile-based partograph is easy to use, easy to interact with, and easy to learn are more likely to perceive it as useful, and consequently, this will lead to a high intention to use it [39].

Another association in this study was that perceived usefulness (β=.184; *P*=.02) positively influenced intention to use. This positive and significant relationship of the construct is in agreement with a study in Turkey on personal digital assistant acceptance (β=.41) [48], a study in Uganda on mobile phone adoption in maternal health care (β=.186) [54], and a study in Ethiopia on eHealth adoption (β=.387) [39]. Thus, obstetric health care providers’ perception of mobile-based partograph usefulness is a valuable predictor of behavioral intention to use. It is important to find out how users measure the usefulness of the technology because the more the obstetric health care providers perceive that a mobile-based partograph improves productivity, performance, and effectiveness, and decreases the duration of recording, the more likely they will intend to use it. Our study shows that perceived ease of use positively (β=.382) and significantly (*P*=.003) influenced attitude toward using electronic partographs. This direct effect of perceived ease of use on attitude is in line with a study in Ethiopia (β=.347) [39]. These findings indicate that if users perceive that the use of electronic partograph is easy, they will develop a positive attitude toward using it, consequently impacting their behavioral intention to use it.

We found that perceived ease of use of mobile-based partographs significantly affected intention to use (β=.369; *P*=.001). This showed that the likelihood of intention to use the mobile-based partograph will increase with an increase in the impression of ease of use. This outcome is consistent with a study on the adoption of health information systems that was conducted in Greece (β=.29) [50] and in Ethiopia (β=.339) [39], demonstrating that requiring less effort will boost the system’s ability to influence people’s intentions to use eHealth systems. Perceived ease of use in this study had the highest path coefficient and a significant impact on usage intention. The more obstetric health care professionals are intentional to use the mobile-based partograph, the less effort it is thought to take to operate it on both a mental and physical level. To meet user expectations, electronic partograph developers should concentrate on the device’s user-friendliness. This might increase the uptake and ongoing use of mobile-based partographs. Additionally, we found that attitudes about adopting mobile-based partographs are influenced by perceived usefulness (β=.521; *P*=.002). This finding is consistent with studies on telemedicine acceptability done in China (β=.43) [85] and Ethiopia (β=.26) [39]. The obstetric health care providers’ attitude toward use was impacted by how much they believed this technology improves performance and productivity in their job. Thus, intention to use is directly influenced by attitude toward use (β=.309; *P*=.002). Our study’s conclusion is consistent with that in a study on the intention of health professionals in Ethiopia to use eHealth (β=.526) and indicates that actions that improve perspectives, such as ongoing training and support and information sharing on eHealth innovations, should be prioritized heavily [39]. The more positive perception the obstetric health care providers developed and had, the higher they intended to use eHealth.

In this study, job relevance significantly and positively (β=.408; *P*=.002) influenced perceived usefulness. This study path relationship is in line with a study that focused on the adoption of technology using modified TAM [44,86]. There should be adequate information provision strategies for end users about the applicability and usefulness of mobile-based partographs in labor management. A study on personal digital assistant acceptance by health care professionals supports this evidence in a way that information provision about the technology applicability by health care institutions promotes technology acceptance [80]. Additionally, job relevance significantly influenced intention to use (β=.185; *P*=.008). This finding is in line with a study conducted on health information technology acceptance [44]. This means that the more probable obstetric health care professionals expected to use a mobile-based partograph, the more they believed it was appropriate, relevant, and vital to their work. Because of this, it is crucial to let obstetric health care professionals know about the use of mobile-based partographs in labor management.

According to this study, subjective norms had an insignificant impact on perceived usefulness (β=.020; *P*=.61). This insignificant influence is in line with the findings of a study on hospital information systems (β=–.18; *P*>.05) [87]. This might be because obstetric health care professionals are more likely to establish their independent judgments and may therefore pay less heed to what other people think. Another factor is that regardless of what is important, others may think all obstetric health care providers will be forced to use mobile-based partographs as long as the government mandates their usage in health care facilities for labor management. Subjective norms also insignificantly influence intention to use (β=–.066; *P*=.07). The result of this study is inconsistent with those of other studies [34,62,75]. Because of the time-consuming and detailed recording in paper-based partographs, obstetric health care providers could record incorrect and incomplete data and they could perform retrograde documentation to avoid accountability. However, in case of the digital partographs, retrograde documentation is not allowed and accountability is assured. To affect the belief that most significant others consider he or she should utilize the mobile-based partograph, the health care system should strengthen the concept of teamwork.

In contrast to a study on the adoption of eHealth in the Amhara region, where attitude was the strongest predictor of intention, in this study, perceived ease of use was the strongest predictor of intention to use [39]. To maximize the likelihood of initial and ongoing usage, mobile-based partographs should be provided with an easy function. The association between job relevance and intention to use as well as the relationship between perceived ease of use and intention to use is partially mediated by perceived usefulness in the study’s proposed model, which is consistent with [88]. However, perceived utility is unable to buffer the link between subjective norms and usage intention.

Perceived ease of use and intention to use were partially mediated by attitude and consistent with the study conducted using modified TAM [83]. Attitude toward using mediates the relationship between perceived usefulness and intention to use. To maximize the benefits of the additional capabilities, makers of eHealth platforms should actively work to change how physicians feel about using them [68]. The proposed modified TAM explained about 85% variance in the obstetric health care providers’ intention to use mobile-based partographs. This shows that the model’s overall predictive ability was high, and the variance in this study was almost equivalent to a study [50] utilizing modified TAM in a health care scenario.

### Limitations of This Study

First, it is difficult to discuss whether the proportion of intention to use mobile-based partographs among obstetric health care providers is high or low due to the lack of similar previous studies. This study was a one-time study. Second, it is difficult to establish a cause-effect relationship in this study due to the cross-sectional nature of this study. Third, as the respondents were only from the government and tertiary care levels, caution must be exercised when applying these findings to all obstetric health care providers in the area.

### Conclusion

In our study, two-thirds of the obstetric health care providers had the intention to use mobile-based partographs. Perceived usefulness, perceived ease of use, job relevance, and attitude positively and significantly influenced their intention to use mobile-based electronic partographs. Among these, perceived ease of use was the strongest potential predictor of intention to use. The connection between exogenous latent variables and intention to use was partially mediated by perceived usefulness and attitude toward usage, except for subjective norms. The modified TAM is an effective model for forecasting the intention of an obstetric health care professional to use a mobile-based partograph.

## Appendix

Multimedia Appendix 1 Supplementary data.

## Abbreviations

AMOS: analysis of moment structure
TAM: Technology Acceptance Model

## Data Availability

The data set used for the analysis in this study can be obtained on request from the corresponding author.
