# Supplementary material for: Intention to Use Mobile-Based Partograph and Its Predictors Among Obstetric Health Care Providers Working at Public Referral Hospitals in the Oromia Region of Ethiopia in 2022: Cross-Sectional Questionnaire Study
Source: Online J Public Health Inform. 2024 May 10;16:e51601. doi: 10.2196/51601 (PMC11127132; doi:10.2196/51601)
Supplement: Multimedia Appendix 1 [file ojphi_v16i1e51601_app1.docx]

**Multimedia Appendix 1**

List of supplementary tables and figures

**Table S1.** Factor loading, composite reliability, average variance extracted, and Cronbach’s alpha of the construct of the proposed model.

| **Constructs** | **Items** | **Factor Loading** | **CR** | **AVE** | **CA** |
| --- | --- | --- | --- | --- | --- |
| **Intention to use** | ITU4  ITU3  ITU2  ITU1 | 0.844  0.904  0.887  0.883 | 0.933 | 0.776 | 0.932 |
| **Subjective Norm** | SN3  SN2  SN1 | 0.902  0.946  0.901 | 0.940 | 0.840 | 0.940 |
| **Job Relevance** | JR3  JR2  JR1 | 0.872  0.897  0.906 | 0.921 | 0.795 | 0.920 |
| **Perceived ease of use** | PEOU4 PEOU3 PEOU2  PEOU1 | 0.871  0.887  0.888  0.843 | 0.927 | 0.761 | 0.926 |
| **Perceived usefulness** | PU4  PU3  PU2  PU1 | 0.890  0.886  0.909  0.914 | 0.945 | 0.810 | 0.944 |
| **Attitude** | AT3  AT2  AT1 | 0.896  0.884  0.907 | 0.924 | 0.802 | 0.924 |

**Table S2.** The result of discriminant validity of the proposed model.

**ITU SN JR PEOU PU AT**

**ITU 0.881**

**SN** 0.622 **0.917**

**JR** 0.830 0.711 **0.892**

**PEOU** 0.861 0.657 0.766 **0.872**

**PU** 0.844 0.636 0.799 0.835 **0.900**

**AT** 0.864 0.674 0.885 0.804 0.826 **0.896**

**Table S3.** The direct, indirect, total effect, types of mediation, and the result of the hypothesis test regarding the obstetrics healthcare provider's intention to use and its predictors.

**Hypothesis Path Effect Significance Mediation Decision**

H11 JR$\to PU \to$ ITU Total Significant Partial Supported

Indirect Significant

Direct Significant

H12 SN$\to PU\to$ ITU Total insignificant No relationship Not supported

Indirect insignificant

Direct insignificant

H13 PEOU$\to PU\to$ ITU Total Significant Partial Supported

Indirect Significant

Direct Significant

H14 PU$\to AT\to$ ITU Total Significant Partial Supported

Indirect Significant

Direct Significant

H15 PEOU$\to AT\to$ ITU Total Significant Partial Supported

Indirect Significant

Direct Significant

***Note:*** *H-hypothesis, AT-attitude, JR-job relevance, PU-perceived usefulness, PEOU-perceived ease of use, SN-subjective norms, ITU-intention to use*

**Table S4.** The result of squared multiple correlations ($R^{2}$) of AMOS 23 output based on data obtained from obstetrics healthcare providers Oromia region Ethiopia 2022.

**Variables** $\boldsymbol{R}^{\boldsymbol{2}}$

Perceived usefulness 0.765

Attitude 0.749

Intention to use 0.854


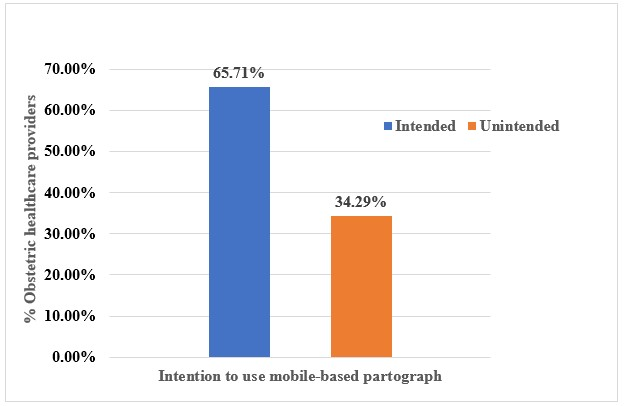


**Figure S1.** The proportion of intention to use mobile-based partograph among obstetric health care providers who were working at public referral hospitals in Oromia region Ethiopia 2022 (n=630).


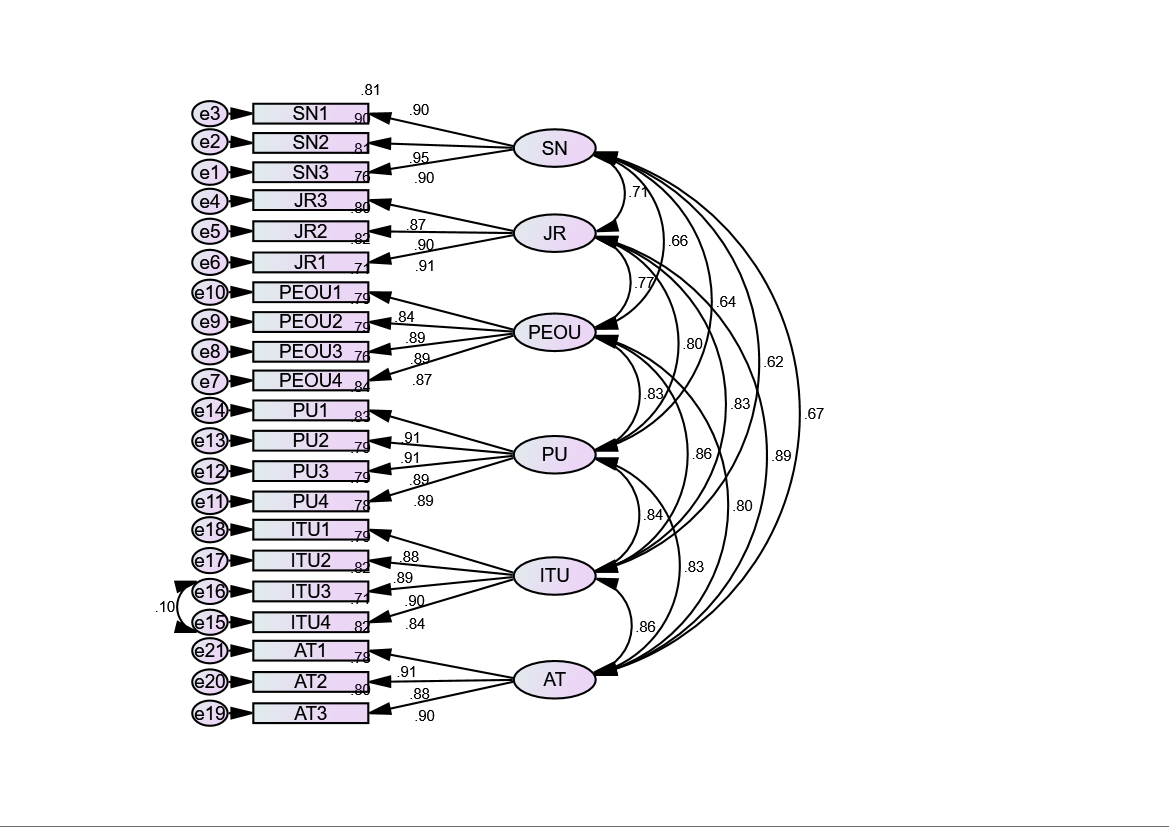


**Figure S2.** Confirmatory factor analysis of standardized estimate of AMOS version 23 output.
